# Supplementary material for: Ecological analysis of Pavlovian fear conditioning in rats
Source: Commun Biol. 2022 Aug 18;5:830. doi: 10.1038/s42003-022-03802-1 (PMC9388582; doi:10.1038/s42003-022-03802-1)
Supplement: Supplementary file 2 — Supplementary Information [file 42003_2022_3802_MOESM2_ESM.pdf]

SUPPLEMENTARY INFORMATION

Ecological analysis of Pavlovian fear conditioning in rats

Peter R. Zambetti[1], Bryan P. Schuessler[1], Bryce E. Lecamp[2], Andrew Shin[3], Eun Joo Kim[1], and Jeansok J. Kim[1]*

[1]Department of Psychology, University of Washington, Seattle, WA 98195

[2]Undergraduate Program in Neuroscience, University of Washington, Seattle, WA 98195

[3]Undergraduate Program in Human Biology, Stanford University, Stanford, CA 94305

*Correspondence

Email: jeansokk@u.washington.edu

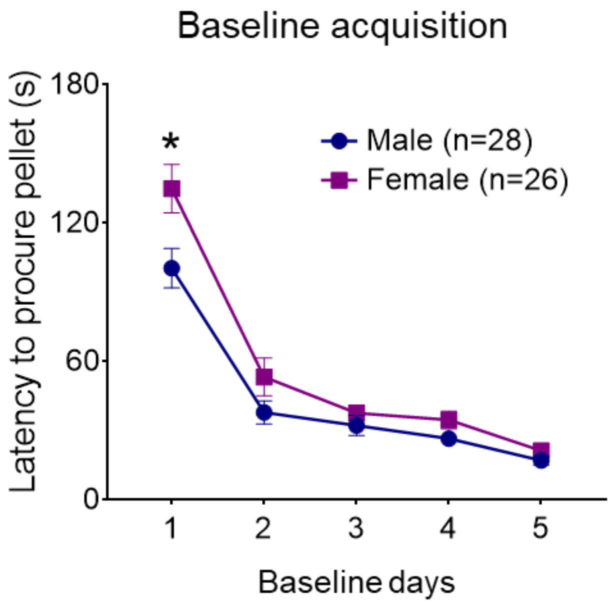

Supplementary Fig. 1: Initial sex differences in the baseline latency to procure pellets. Average latencies (±SEM) to procure food pellets in foraging area. Females had longer latencies to procure pellets than males during the first baseline session day 1 (Mann-Whitney U, z = 2.476, p = 0.013) but not subsequent baseline session days 2-5 (Mann-Whitney U, Baseline 2: z = 1.039, p = 0.299; Baseline 3: z = 1.922, p = 0.055; Baseline 4: z = 1.112, p = 0.266; Baseline 5: z = 1.904, p = 0.057). * $p < 0.05$.

a

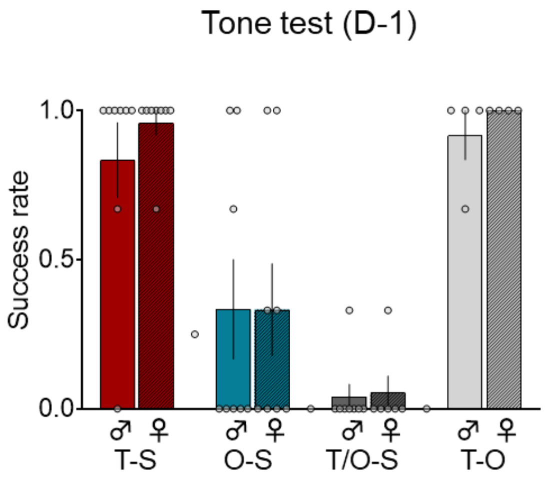

b

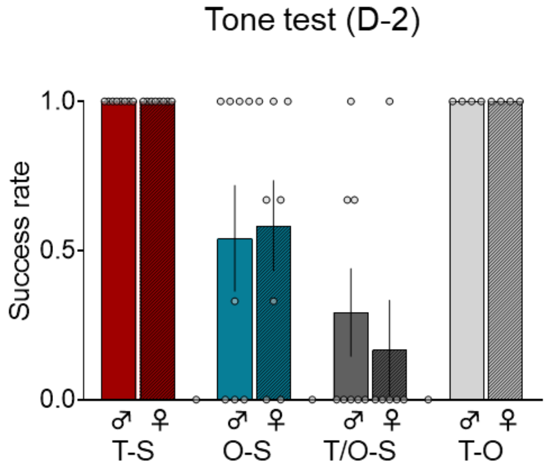

Supplementary Fig. 2: No reliable sex differences in the procurement of pellets during tone tests. **a,** Mean (± SEM) success rate for procuring food pellets during the first day of tone testing. No significant differences were found between males and females in tone-shock (T-S), owl-shock (O-S), tone/owl-shock (T/O-S) and tone-owl (T-O) groups (Mann-Whitney U; $z = 0.694$, $p = 0.645$ for T-S; $z = 1.0$, $p = 0.317$ for O-S; $z = 0.212$, $p = 1.0$ for T/O-S; $z = 0.234$, $p = 0.815$ for T-O). **b**, Mean (± SEM) success rate for procuring food pellets during the second day of tone testing. No sex differences were observed in all groups (Mann-Whitney; $z = 0$, $p = 1.0$ for T-S; $z = 0.056$, $p = 0.955$ for O-S; $z = -0.649$, $p = 0.662$ for T/O-S; $z = 0$, $p = 1.0$ for T-O).

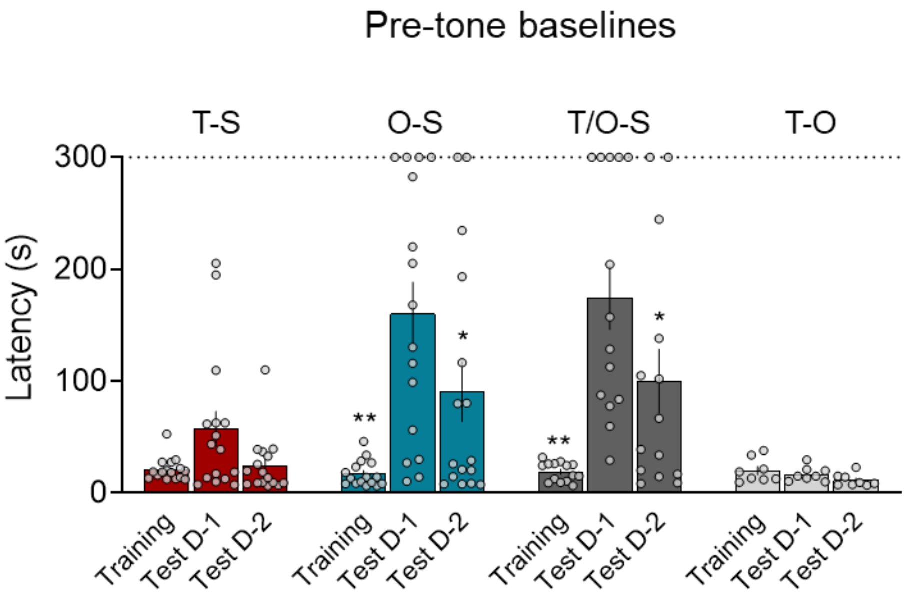

Supplementary Fig. 3: Comparisons of latencies to procure pellets during pre-fear conditioning baseline and pre-tone testing baseline days 1 and 2. The baseline latencies to procure pellets prior to the fear conditioning session (Fig. 2a) were not statistically different from the day 1 (Fig. 3a) and day 2 (Fig. 3c) pre-tone test baseline latencies after the fear conditioning session in both tone-shock (T-S) and tone-owl (T-O) paired animals (Related-samples Wilcoxon signed rank test; Baseline vs. D-1: $z = 1.293$, $p = 0.196$ for T-S; $z = -0.560$, $p = 0.575$ for T-O; Baseline vs. D-2: $z = -0.155$, $p = 0.877$ for T-S; $z = -1.82$, $p = 0.069$ for T-O). This indicates that neither the tone-shock group nor the tone-owl group showed evidence of contextual fear conditioning. However, the O-S and T/O-S groups pre-tone test baseline latencies were significantly longer than the training day baseline latencies (Related-samples Wilcoxon signed rank test; Baseline vs D-1: $z = 3.517$, $p < 0.001$ for O-S; $z = 3.296$, $p = 0.001$ for T/O-S; Baseline vs D-2: $z = 2.095$, $p = 0.036$ for O-S; $z = 2.542$, $p = 0.011$ for T/O-S), suggesting the O-S and T/O-S groups did form a contextual fear memory of the foraging arena. * $p < 0.05$, ** $p < 0.01$

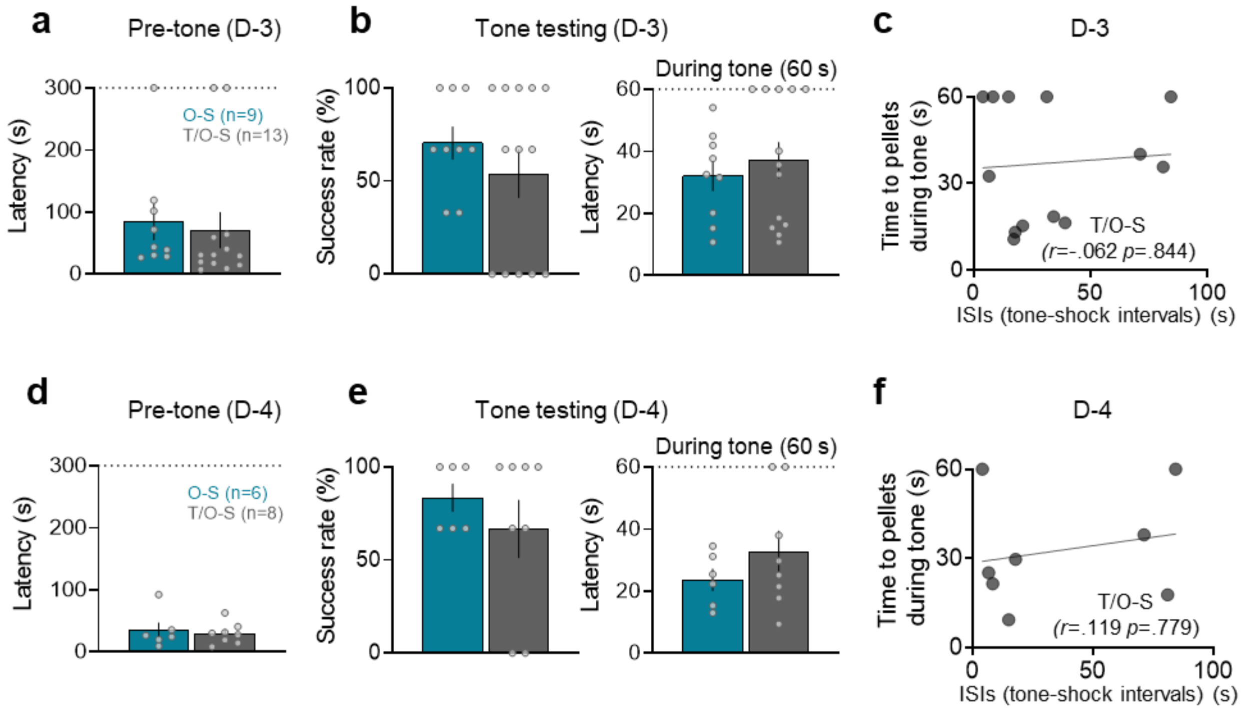

Supplementary Fig. 4: Extended tone testing information. Analyses of the third testing day (D-3) revealed there were no significant differences between the O-S and T/O-S groups in **a,** the mean latency (± SEM) to procure the pellet during the pre-tone baseline trials (Mann-Whitney U, z = 1.304, p = 0.209); **b,** the mean success rate and the latency to procure the pellet during the tone trial (Success Rate: Mann-Whitney U, z = 0.63, p = 0.528; Tone-Pellet Latency: Mann-Whitney U, z = -0.638, p = 0.523); and **c,** the correlation (Spearman's correlation coefficient) between the latency to procure the pellet during the tone and the time interval between the tone CS onset and shock onset (ISIs) during training. Same analyses of the fourth testing day (D-4) showed there were no significant group differences in **d, t**he mean latency (± SEM) to procure the pellet during the pre-tone baseline trials (Mann-Whitney U, z = 0, p = 1.0); **e,** the mean success rate and the latency to procure the pellet during the tone trial (Success Rate: Mann-Whitney U, z = 0.425, p = 0.671; Tone-Pellet Latency: Mann-Whitney U, z = -0.646, p = 0.518); and **f,** the correlation between the latency to procure the pellet during the tone and the time interval between the tone CS onset and shock onset (ISIs) during training.

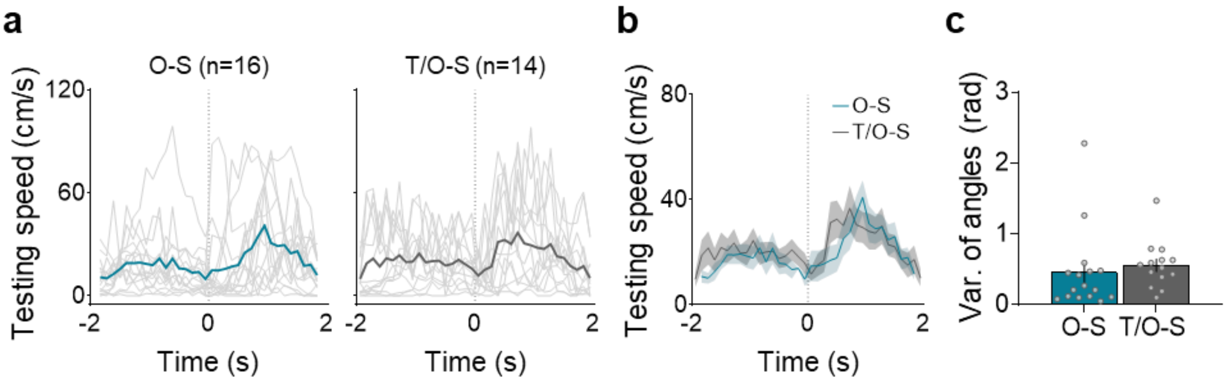

Supplementary Fig. 5: Instantaneous speeds during tone testing and angle trajectories of escape. **a,** Mean (± SEM) speed of each rat as it approached and triggered the tone during the tone testing day. **b,** No significant difference was found between the speeds of the O-S and T/O-S groups (Mann-Whitney U, z = -1.539, p = 0.124). **c,** No significant differences were found between the O-S and T/O-S groups' return trajectories, i.e., the variance of angles (radians), to the nest during the tone testing (Mann-Whitney U, z = -1.871, p = 0.064).

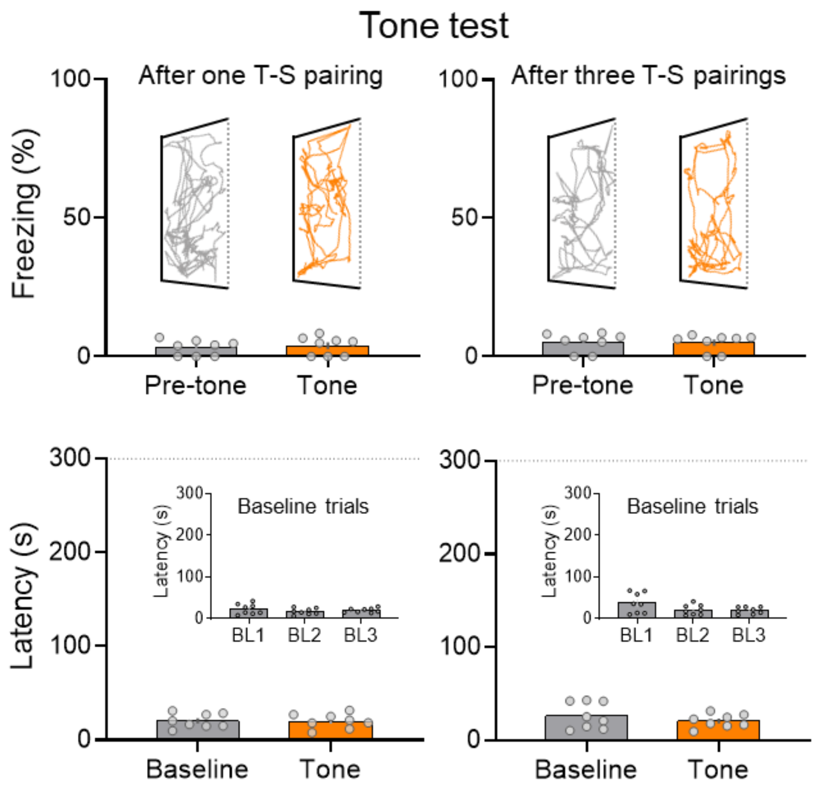

Supplementary Fig. 6: Tone testing inside the nest and latency. Top, Mean (+ SEM) and individual freezing data during the 1 min pre-tone and 1 min tone periods in the nest with the gateway closed. After a single tone-shock pairing (left), there was no significant increase in freezing from the pre-tone period to the tone period (Related-samples Wilcoxon signed rank test; z = 0.524, p = 0.6). After a total of three tone-shock pairings (right), there was still no reliable increase in freezing from the pre-tone period to the tone period (Related-samples Wilcoxon signed rank test; z = -0.140, p = 0.889). Insets show representative track plots of an animal inside the nest during the pre-tone and tone periods after one and three tone-shock pairings. **Bottom,** Mean (+ SEM) and individual latencies to procure the food pellet during the baseline and tone foraging trials. After a single tone-shock pairing (left), there was no significant difference in the procurement latency before and during the tone (Related-samples Wilcoxon signed rank test; z = -0.560, p = 0.575). After a total of three tone-shock pairings (right), again there was no difference in the procurement latency before and during the tone (Related-samples Wilcoxon signed rank test; z = -1.680, p = 0.093). Insets show the three baseline trials.

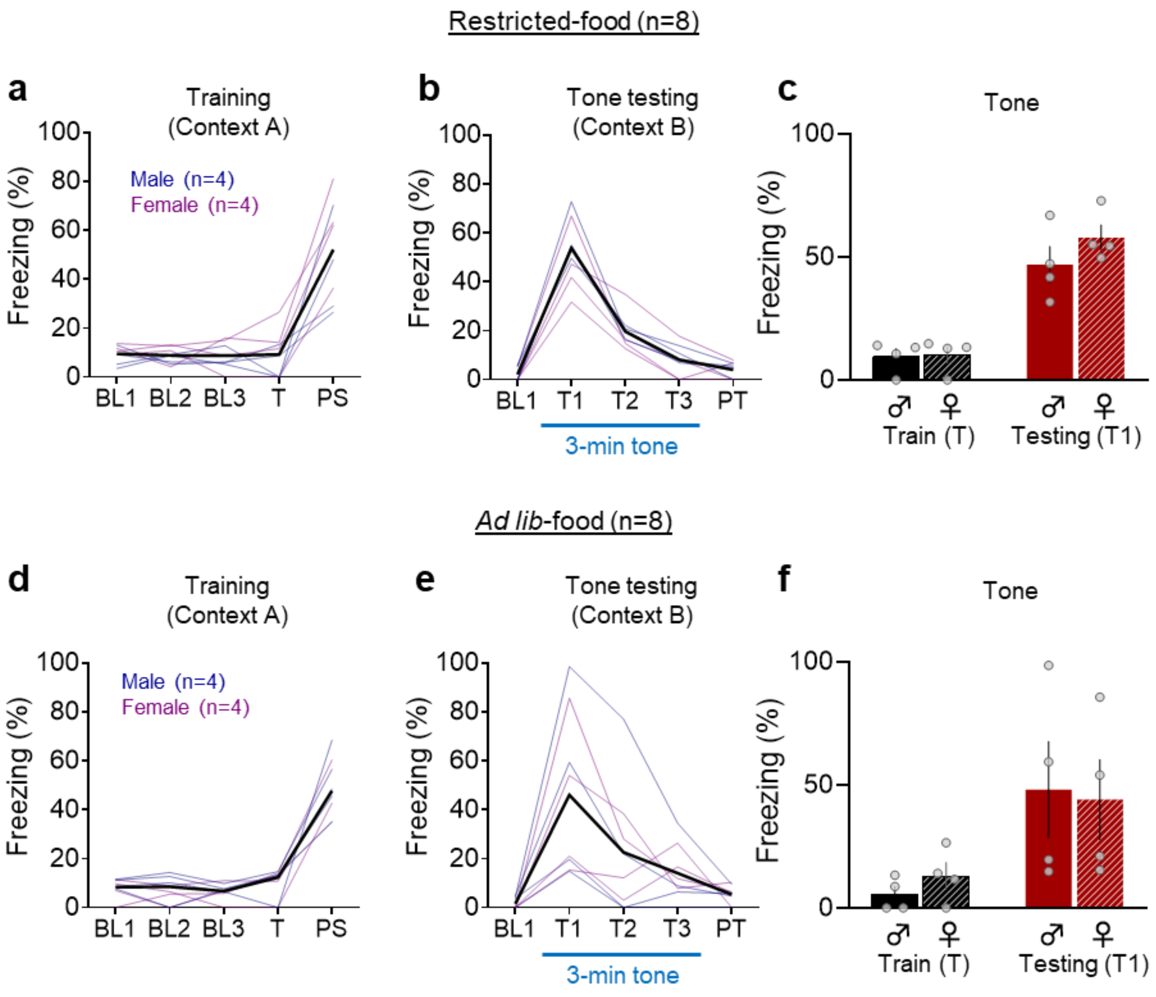

Supplementary Fig. 7: No sex differences during fear conditioning in a standard experimental chamber. (restricted-food) a, During a single-trial CS-US training session there were no significant differences in freezing between male and female rats (Mann-Whitney U test, BL1: z = -0.866 , p = 0.486; BL2: z = -1.307, p = 0.200; BL3: z = -0.862, p = 0.386; T: z = -0.436, p = 0.686; PS: z = -0.577, p = 0.564). **b**, The following day rats were placed in a new context and presented with the same tone and freezing was measured. There were no significant differences between males and females' freezing levels during the tone presentation (Mann-Whitney U test, BL1: z = -0.661, p = 0.508; T1: z = -1.443, p = 0.2; T2: z = -1.155, p = 0.343; T3: z = -0.581, p = 0.686; PT: z = 0.296, p = 1.0). **c**, Mean (± SEM) percent freezing during tone exposure for the Training (T) and Testing (T1; during the first min) sessions. There were no significant sex differences in freezing during either tone exposure (Mann-Whitney U test, Train: z = -0.436, p = 0.686; Testing: z = -1.443, p = 0.2). **(*ad lib*-food) d,** During a single-trial CS-US training

session there were no significant differences in freezing between male and female rats (Mann-Whitney U test, BL1: $z = -1.307$, $p = 0.2$; BL2: $z = -1.155$, $p = 0.343$; BL3: $z = -0.872$, $p = 0.486$; T: $z = -1.183$, $p = 0.343$; PS: $z = -1.155$, $p = 0.343$). **e**, The following day rats were placed in a new context and presented with the same tone and freezing was measured. There were no significant differences between males and females' freezing levels during the tone presentation (Mann-Whitney U test, BL1: $z = 1.984$, $p = 0.114$; T1: $z = 0.145$, $p = 1.0$; T2: $z = -0.581$, $p = 0.686$; T3: $z = -0.866$, $p = 0.486$; PT: $z = -0.726$, $p = 0.486$). **f**, Mean (± SEM) percent freezing during tone exposure for the Training (T) and Testing (T1; during the first min) sessions. There were no significant sex differences in freezing during either tone exposure (Mann-Whitney U test, Train: $z = -1.183$, $p = 0.343$; Testing: $z = 0.145$, $p = 1.0$).

Supplementary Table 1

Groups	T-S (8 males, 8 females)				O-S (8 males, 8 females)				T/O-S (8 males, 6 females)				T-O (4 males, 4 females)			
Measures	M, Med (IQR)	F, Med (IQR)	*z*	*p*	M, Med (IQR)	F, Med (IQR)	*z*	*p*	M, Med (IQR)	F, Med (IQR)	*z*	*p*	M, Med (IQR)	F, Med (IQR)	*z*	*p*
Baseline latency to pellet (s)	15 (12.8,20.2)	20.5 (18.4, 29.2)	1.472	0.161	14.2 (9.8, 26.1)	9.6 (8.3,30.1)	-0.473	0.645	20.2 (11.2, 27.5)	19.8 (8.3, 25.4)	-0.582	0.573	17.3 (12.3, 30.8)	15.8 (10.4, 33.3)	-0.289	0.886
Latency to Leave Nest (s)	18.9 (2.7, 66.5)	7.1 (2.4, 26)	-0.840	0.442	2.4 (1.8, 4.4)	1.9 (.5, 8.3)	-0.316	0.798	27.7 (17.3, 58.3)	10.7 (8.1, 31.6)	-1.420	0.181	7.3 (4.5, 14.9)	24.9 (8.9, 45.7)	1.307	0.200
Latency to Trigger Zone (s)	8.7 (5.5,11.9)	7 (4.6, 11.2)	-0.735	0.505	3.9 (1.1, 5.6)	7.9 (1.8, 10.9)	1.155	0.279	7.1 (3.6, 9.9)	5.3 (4.6, 7.3)	-0.775	0.491	6.5 (5.9, 14.1)	9.2 (6.3, 13.1)	0.581	0.686
Escape Latency to Nest (s)	2.9 (1.9, 5)	2.9 (1.5, 3.3)	-0.578	0.574	1.7 (1.1, 2.0)	2.7 (2, 3.6)	2.052	0.040	1 (0.8, 2.3)	1.8 (1.1, 1.9)	0.780	0.491	5.9 (2.6, 7.1)	0.9 (0.7, 1.6)	-2.021	0.057
Escape Distance (cm)	105.8 (99.2, 111.9)	109.9 (103.1, 130.7)	1.155	0.279	131.9 (117.6, 140.3)	142.5 (121.8, 158.5)	0.735	0.505	117.6 (111.1, 131.5)	125.8 (120.4, 130.1)	0.387	0.755	106.5 (99.8, 124.1)	104.5 (99.1, 108.8)	-0.577	0.686
Variance of Angles	0.19 (0.1, 0.8)	0.62 (0.3, 1.5)	1.575	0.130	1.7 (1.1, 2.1)	2.5 (1.3, 2.9)	1.890	0.059	1.5 (0.2, 2.6)	1.6 (1.1, 2.1)	0.129	1.000	0.3 (0.05, 0.5)	0.2 (0.1, 0.3)	-0.578	0.686
Latency to pellet (Pre-tone), D-1 (s)	13.2 (8.3, 156)	56.5 (24.9, 62.7)	1.471	0.161	160.7 (18.3, 300)	149.2 (67, 267)	0.053	1.000	122.5 (79.2, 300)	166.5 (91.8, 300)	0.264	0.852	15 (13.8, 18.8)	15.7 (10.1, 27.5)	0.000	1.000
Latency to Leave Nest, D-1 (s)	20.6 (7.6, 25.7)	9.1 (4.6, 17.6)	-1.575	0.130	41.8 (11.9, 242.2)	24.9 (7.9, 151.4)	-1.155	0.279	36.7 (23.1, 77.1)	95.3 (43.8, 188.3)	1.291	0.228	3.2 (2.8, 6.7)	3.2 (1.8, 3.6)	0.000	1.000
Latency to Trigger Tone, D-1 (s)	9.6 (2.7, 25.7)	3.9 (2.1, 11.5)	-0.840	0.442	12.3 (7, 22.5)	12.1 (5.3, 48.4)	0.192	0.902	24.8 (10.3, 32.7)	62.6 (22.5, 99.7)	1.512	0.164	9.8 (6.5, 19.4)	9.5 (7.7, 12.3)	0.146	1.000
Latency to pellet (Tone), D-1 (s)	15.9 (5.3, 4.3)	9.6 (5.6, 18.3)	-0.630	0.574	60 (21.7, 60)	52.3 (31.7, 60)	-0.231	0.878	60 (60, 60)	60 (56.5, 60)	-0.318	0.852	5.8 (4.4, 26.6)	4.9 (3.2, 5.3)	-0.577	0.686
Latency to pellet (Pre-tone), D-2 (s)	19.2 (7.6, 34.2)	11.7 (9, 37.5)	0.105	1.000	27.5 (11.1, 224.3)	50.5 (10.2, 107.6)	-0.263	0.798	27.2 (10.4, 93.2)	121.7 (33.5, 258.5)	1.616	0.108	8.2 (7.2, 13.4)	11.8 (7.8, 20.8)	0.866	0.486
Latency to Leave Nest, D-2 (s)	6.7 (4.9, 6.9)	4.7 (3.2, 9.1)	-1.051	0.328	9.4 (4.2, 46.9)	20 (4.9, 30.2)	0.210	0.878	17 (14.3, 31.6)	47.1 (10.4, 186.9)	0.775	0.491	3.1 (1.5, 5.2)	4 (2.4, 6.3)	0.866	0.486
Latency to Trigger Tone, D-2 (s)	5.7 (3.5, 9.1)	1.7 (0.6, 7.9)	-1.471	0.161	9.5 (1.5, 57.9)	6.8 (2.5, 19.7)	-0.579	0.613	17.7 (5.9, 32.5)	25.8 (13.9, 40.9)	0.878	0.435	4.9 (1.9, 12.3)	3.8 (2.8, 9.7)	0.000	1.000
Latency to Pellet (Tone), D-2 (s)	6.3 (5.6, 9.8)	4.7 (3.8, 8.1)	-1.682	0.105	35.8 (9.8, 60)	39.5 (15.5, 56.9)	0.160	0.878	60 (31.6, 60)	60 (46.7, 60)	0.566	0.662	4.1 (3.5, 6.1)	4.6 (3.3, 5.9)	-0.290	0.772
Success rate (%)	100 (71, 100)	100 (100, 100)	1.243	0.382	28.5 (0.0, 87.5)	33 (8.3, 8.5)	0.270	0.798	0 (0, 45.7)	0 (0, 16.7)	-0.567	0.662	100 (87.3, 100)	100 (100,100)	1.000	0.686
Trials to Success (count)	1 (1,1)	1 (1,1)	0.091	1.000	4.5 (1.3, 7.0)	3.5 (1.5, 6.3)	-0.535	0.645	7 (4.3, 9.8)	9.5 (5.8, 16)	1.171	0.282	1 (1, 1.8)	1 (1, 1)	-1.000	0.686

Supplementary Table 1. Male (M) and female (F) median (Med) with interquartile range (IQR) in each experimental group and across all the behavioral measures presented in the main text. There were no significant differences between male and female rats in all the measures except for the Escape Latency to Nest after owl-shock pairing in the O-S group (Mann-Whitney U tests; *z* and *p* values).
