# Supplementary material for: Ecological analysis of Pavlovian fear conditioning in rats
Source: Commun Biol. 2022 Aug 18;5:830. doi: 10.1038/s42003-022-03802-1 (PMC9388582; doi:10.1038/s42003-022-03802-1)
Supplement: Supplementary file 3 — Description of Additional Supplementary Files [file 42003_2022_3802_MOESM3_ESM.pdf]

Description of Additional Supplementary Files

File name: Supplementary Movie 1
Description: Representative foraging and escape behaviors of a rat presented with an owl-shock pairing. As the animal comes near a pellet, it encounters a swooping owl (from behind a black curtain) followed by a dorsal neck/body shock pain. The rat flees to the nest without procuring the pellet.

File name: Supplementary Movie 2
Description: The next day, as the same O-S rat advances towards a pellet, a novel tone is presented for the first time. In response to the tone, the rat promptly flees to the nest without procuring the pellet.
